# Influence of Organic and Inorganic Fertilizers on Tea Growth and Quality and Soil Properties of Tea Orchards’ Top Rhizosphere Soil

**DOI:** 10.3390/plants13020207

**Published:** 2024-01-11

**Authors:** Manzoor, Lifeng Ma, Kang Ni, Jianyun Ruan

**Affiliations:** 1 Graduate School, Chinese Academy of Agricultural Sciences, Beijing 100081, China; manzoorali3755@gmail.com; 2 Key Laboratory of Tea Biology and Resource Utilization of Tea, Tea Research Institute, Chinese Academy of Agriculture Sciences, The Ministry of Agriculture, Hangzhou 310008, China; 3 Key Laboratory of Tropical Forest Ecology, Xishuangbanna Tropical Botanical Garden, Chinese Academy of Sciences, Xishuangbanna 666303, China

**Keywords:** tea plant growth, chlorophyll, integrated fertilization, amino acids, catechins, macro and micronutrients, soil properties

## Abstract

Organic-based fertilizers have been ratified to be effective in ameliorating tea growth and the fertility of soil. However, the effect of integrated fertilization on tea growth and quality and the chemical properties of the soil in tea gardens are unclear. To address this, from 2020 to 2021, five different treatments were carried out in the greenhouse of the Tea Research Institute, Hangzhou, CAAS, including CK (control), NPK (chemical fertilizers), RC (rapeseed cake), NPK+B (chemical fertilizer + biochar), and NPK+RC, to investigate the effects of different fertilizations on soil chemistry and tea growth and quality. The results indicated that NPK+B and NPK+RC significantly improved the different amino acid and catechin concentrations in the young shoots, stems, and roots of the tea compared to the CK. The plant growth parameters, e.g., the plant height, no. of leaves, mid-stem girth, and fresh weights of stems and leaves, were significantly increased with integrated fertilization (NPK+B and NPK+RC) compared to the CK and solo organic and inorganic fertilizers. The chlorophyll contents (Chl a, Chl b, and Chl a+b) were generally higher with NPK+RC than with the CK (37%, 35%, and 36%), RC (14%, 26%, and 18%), and NPK (9%, 13%, and 11%) treatments. Integrated fertilization buffered the acidic soil of the tea garden and decreased the soil C:N ratio. NPK+RC also significantly increased the soil’s total C (31% and 16%), N (43% and 31%), P (65% and 40%), available P (31% and 58%), K (70% and 25%), nitrate (504% and 188%), and ammonium (267% and 146%) concentrations compared to the CK and RC. The soil macro- (Mg and Ca) and micronutrients (Mn, Fe, Zn, and Cu) were significantly improved by the RC (100% and 72%) (49%, 161%, 112%, and 40%) and NPK+RC (88% and 48%) (47%, 75%, 45%, and 14%) compared to the CK. The chlorophyll contents and soil macro- and micronutrients were all significantly positively correlated with tea quality (amino acids and catechin contents) and growth. These results indicated that integrated fertilization improved the soil nutrient status, which is associated with the improvement of tea growth and quality. Thus, integrated nutrient management is a feasible tool for improving tea growth, quality, and low nutrient levels in the soil.

## 1. Introduction

After water, tea is the second-most popular non-alcoholic beverage in the world. Tea is made from the fresh leaves of a tea tree [1,2]. Tea is a very important cash crop that is largely cultivated in tropical and sub-tropical regions, such as Japan, China, Vietnam, and India [3]. Over the past few decades, the popularity of tea has grown steadily due to its abundance of beneficial amino acids, vitamins, and antioxidants [4]. Tea contains many secondary metabolites that are closely related to its quality, such as tea alkaloids, tea polyphenols, and free amino acids, which are rich in aroma, flavor, and health benefits [5]. In general, the umami taste of tea is caused by free amino acids, especially theanine, and as the amino acid concentration increases, the intensity of the flavor is augmented. Theanine is the most abundant amino acid in tea, typically accounting for 60% to 70% of the total free amino acid content in tea and about 0.2% to 2% of the dry weight of tea [6,7]. Tea polyphenols, such as catechins, are one of the principal contributors to tea’s astringent properties [1,8]. The catechins in tea are not only responsible for the ultimate organoleptic properties, but they are also closely linked to many health benefits, such as anti-cancer [9], anti-inflammatory [10], and antibacterial effects [11]. Numerous research studies have shown that catechins are abundant in green tea, accounting for about 30% of the dry weight of green tea leaves [12]. Catechins are flavan-3-alcohols that occur naturally in tea. Epicatechin and its gallate derivatives are the main flavan-3-ols in green tea [13]; EGCG, the main catechin, accounts for more than 10% of green tea leaves’ dry weight [14,15]. Furthermore, some research studies have shown that gallate-type catechins are more bitter and astringent than free catechins. The concentrations of compounds affecting tea quality vary significantly due to cultivation practices and growing environments (soil, climate, and altitude) [16,17]. As the demand for tea production continues to grow, farmers are increasingly using fertilizer to maximize tea production [18]. All the same, many research studies have shown that the long-term overuse of chemical fertilizers in tea cultivation can lead to soil degradation problems, such as a reduction in beneficial bacteria, nutrient loss, structural disruption, and acidification [19,20]. Tea plants prefer acidic soils, and the optimum pH for tea growth ranges from 4.5 to 5.5 [21]. However, in a nationwide soil survey of major tea-growing regions in China, about 46% of the tea soil samples had a pH of less than 4.5 [22]. Due to these issues, the quality and yield of green tea were reduced, leading to proven limitations in sustainable tea production [23]. Therefore, there is an acute need to adjust and optimize fertilization strategies to ameliorate soil fertility and tea garden traits, thereby improving tea quality and yield.

It is established that applying organic amendments is the best option for mitigating the negative effect of mineral fertilizer by improving organic carbon and soil porosity [24]. Organic fertilizer has the advantages of a negative surface charge, high porosity, higher alkalinity, and higher carbon content [25]. Over the past decade, many researchers have delineated the benefits of organic fertilizers on soil physicochemical characteristics, such as increasing the soil pH, reducing the bulk density, and improving the nutrient availability [26,27]. Furthermore, organic fertilizers have been shown to improve soil enzyme activities and alter the community composition [28,29]. However, due to the limited nutrient contents of organic fertilizers, their impact on crop yields is not particularly pronounced [30]. Therefore, the combined use of organic and inorganic fertilizers has received more advertence recently [31,32]. The combined application of chemical and organic fertilization was more effective than organic fertilizers alone in improving soil nutrients and yield. For example, N fertilizer plus biochar improves the soil nutrient availability and rainfed rice yields while decreasing soil NO_3_^−^ leaching [33]. Faloye et al., 2019 [34] indicated that the application of chemical and organic fertilization increased the nutrient uptake in maize and soil nutrients compared to organic or inorganic fertilizers alone. Although integrated fertilization applications have been reported in several crops, vegetables, and medicinal plants [35,36,37], few research studies were carried out to investigate the effects of organic and mineral fertilizers on tea growth and quality and improved soil properties in tea gardens.

Despite the growing understanding of the importance of organic fertilizers, previous research has focused on improving crop yields and soil nutrients [33,38]. Little is known about the effect of integrated fertilization on soil characteristics and its role in improving plant growth and quality. Particularly in highly acidic soils, such as tea garden soils, organic fertilizers play an important role in soil OM turnover. [24,39]. The influence of integrated fertilization on soil properties and its role in improving tea growth and quality are unclear. A comprehensive understanding of the impact of organic fertilizers on tea growth, quality, and soil characteristics may provide new insights into improving tea production, quality, and soil management practices in tea gardens.

In this greenhouse experiment, five treatments (CK, NPK, NPK+B, NPK+RC, and RC) were used for greenhouse experiments to examine (1) the influence of different chemical and organic fertilization’s on tea growth and quality, (2) the changes in soil chemical properties due to different fertilizer management methods, and (3) the effect of soil chemical properties on tea quality and growth. We also assume that using organic or organic-based fertilizers changes soil chemistry. We also hypothesize that changes in tea quality and growth correlate with changes in soil chemical properties.

## 2. Materials and Methods

### 2.1. Experimental Site

**Figure 1:**
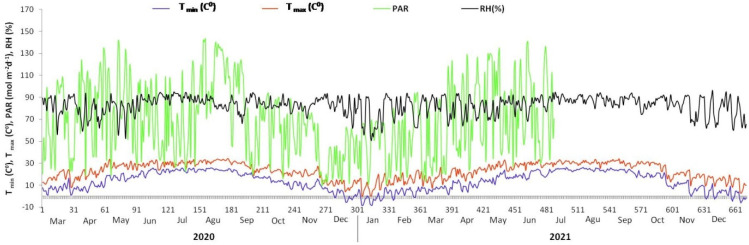
Weather data during entire experimental period from 2020 to 2021. PAR = Photosynthetic Active Radiation (mol m^−2^ d^−1^); T_max_ (C°) = Maximum Temperature; T _min_ (C°) = Minimum Temperature; RH (%) = Relative humidity.

The greenhouse experiment was carried out in the 2020–2021 period at the Tea Research Institute, CAAS (30°10′ N, 120°05′ E, 22 m MSL), situated in the main green tea growing area in China. The research site has a humid sub-tropical climate with an average RH of about 70%. The average maximum temperature is 22.8 °C (73 °F), and the average minimum temperature is 13.5 °C (56.3 °F) (Figure 1). According to the GSCC, research soils are classified as red-yellow soils, equivalent to Alfisol in the World Reference Base for Soil Resources [40,41], taken from the garden of Royal Tea Village Co., Ltd., Shaoxing, China, 20 cm deep (top rhizosphere soil). According to Bao 2005 [42], properties of the top rhizosphere soil of the tea garden were determined before the experiment began (Table 1).

### 2.2. Seedling Establishment and Treatment Design

Tea seedlings germinate from the seed of the tea variety “Longjing-43” [43]. The soil for the greenhouse experiment was taken from a tea garden and air dried, sifted through a 2 mm sieve, and then thoroughly mixed, and 4.6 kg of soil was loaded into pots (14 × 24 cm diameter and depth) with 3 cm of perlite at the bottom. In March 2020, 3 tea seedlings were planted in small pots. After one month, healthy seedlings were left, and the remaining seedlings were discarded. In October 2020, the tea plant was transplanted into large pots.

An amount of 8 kg of soil was placed into a pot (25 cm inner diameter × 25 cm depth). Experimental treatments included control (CK) (no fertilizers), 100% rapeseed cake (RC), 100% NPK, 50% NPK + 50% rapeseed cake (NPK+RC), and 100% NPK + biochar (NPK+B).

NPK was used at a ratio of 75:10:25 mg per kg of soil. The application doses of rapeseed cake and biochar were 1.26 and 1.25 g per kg soil, respectively. KH_2_PO_4_ (39.09% K_2_O and 30.97% P_2_O_5_) was used as a compound fertilizer for P and K, Urea (46% N) was used as N fertilizer, and potassium chloride (KCl) was used as K fertilizer (60% K_2_O). Rice straw was heated for 2 h at 500 °C with limited O_2_ to make biochar. The nitrogen, phosphorus, and potassium contents of RC were about 5.98%, 3.42%, and 1.96%, respectively. Biochar and RC were amalgamated with soil and loaded into pots. Urea, KH_2_PO_4_, and KCl solutions were prepared and applied in three split doses at a 2-week interval before transplanting in the July–August 2020 period and 62 days after transplanting from December 2020 to January 2021. A 30% (*w*/*w*) soil moisture was maintained. A completely random design with four replicates (CRD) was exploited for the treatment arrangements. Plant, root, and soil samples were collected in October 2021. The research duration was 480 days.

### 2.3. Determination of Different Amino Acid and Catechin Contents by HPLC

We weighed the milled sample to obtain 250 mg and added 1.5 mL of a 75% methanol extract solution to the Eppendorf tube. We shook the tube with a vortex shaker and placed it in an ultrasonic shaker water bath for 15 min, shook it up and down twice at 7 min intervals, and then centrifuged at 11,000 rpm at 25 °C for 10 min. We filtered the extract through a 0.22 μm PTFE filter and divided the sample solution into two parts for catechin and amino acid analysis in a DRAM glass tube.

The e2695-connected and 2998-Photodiode Array Detector System (Waters) was used for high performance liquid chromatography analysis, which injected 25 μL and 10 μL of free amino acid and catechin sample solutions, respectively. For catechins, mobile phase A consisted of ddH_2_O containing 2% formic acid, while mobile phase B consisted of acetonitrile (ACN), a high-performance liquid chromatography solvent (Sigma-Aldrich Co., St. Louis, MO, USA). Sample was eluted at a flow rate of 1 mL/min with a column temperature of 40 ± 1 °C and monitored at 278 nm. For amino acids, mobile phase A had AccQ. Tag eluent of water, while mobile phase B was acetonitrile, and the column temperature was set to 37 ± 2 °C. The rest of the process was carried out according to the AccQ. Label Chemical Packaging Instruction Manual. We compared the retention time of the sample with the standard, and identified the peak of catechins and amino acids, as described in the manual.

### 2.4. Determination of Chlorophyll

With minor modifications, chlorophyll (Chl) contents were measured according to the protocol of Ni et al., 2009 [44]. We placed the fresh leaves in a mortar and ground them into a fine powder. We added 400 mg of sample and 5 mL of 80% acetone to a 15 mL falcon tube, mixed it for 5 min, and then kept it at 4 °C for 15 min in the dark (chlorophyll is hydrolyzed under light). Then, we centrifuged the mixture at 3000 rpm for 15 min, transferred the supernatant to a new centrifuge tube, and protected it from light. This process was repeated twice. We combined the supernatant and obtained a total volume of 25 mL with 80% acetone. We shook the solution thoroughly and determined the absorbance of Chl using a spectrophotometer (varian carries winning UV) with 80% acetone as a blank (control). The Chl contents were measured using the following equation:

$${Chlorophyll\;a}_{\left(mg/g\right)}=\frac{[{12.7\times A}_{663}-{2.69\times A}_{645}]\times V}{1000\times W}\;{Chlorophyll\;b}_{\left(mg/g\right)}=\frac{[{22.9\times A}_{645}-{4.68\times A}_{663}]\times V}{1000\times W}\;{Chlorophyll\;a+b}_{\left(mg/g\right)}=Chlorophyll\;a+Chlorophyll\;b\;Chlorophyll\;a/b\;ratio=\frac{Chlorophyll\;a}{Chlorophyll\;b}$$

where W = sample weights (g); V = volume of supernatant (ml); A_663_ = 663 nm wavelength absorbance; and A_645_ = 645 nm wavelength absorbance.

### 2.5. Plant Growth Parameters

We randomly selected 4 potted plants from each treatment unit, cut off the shoots at the level of the crowns (the point of separation between the root system and the stem), plucked off all leaves (counted), and separated the plant into leaves, stems, and roots. We measured the plant height with a ruler and stem diameter with a digital caliper. We recorded the fresh weights of leaves and stems (cut stem into 7–8 cm parts). The young shoots (1 bud and 2 leaves), stems, and roots were dried, grinded, and passed through a mesh sieve with a 0.7–1.0 mm size. Before analysis, we stored the powdered young shoots, stems, and roots in a zipper-lock bag.

### 2.6. Soil Macro^−^ and Micronutrients

In late October 2021, soil samples were collected. We mixed the soil from the same treatment pots into one sample and removed any stones, plant residues, and roots by hand. The collected fresh soil sample was passed through a 2 mm sieve and divided into two parts. Portions of the mineral nitrogen (NO_3_^−^ and NH_4_^+^) were immediately analyzed, and the other part was air-dried before other soil properties were determined.

Orion 3 STAR pH meter (Thermoer Inc., Boston, MA, USA) was used to measure soil pH at a ratio of 1:1 (*w*/*v*) in double-distilled water (ddH_2_O). A C/N elemental analyzer (Vario Max, Elementar, Frankfurt, Germany) was used to measure soil’s total N and C contents. Available soil nutrients (K, P, Ca, Fe, Mg, Mn, Zn, and Cu) were extracted from the soil using the Mehlich-3 method (Mehlich, 2008), and their contents were determined using ICP-AES (Thermo Jarrell Ash Ltd., Franklin, MS, USA). TP and TK were determined by digestion. After digestion, the sample was diluted in a 50 mL volumetric flask with 50 mL of ddH_2_O, and the contents of TP and TK were measured using ICP-AES (Thermo Jarrell, Ash. Ltd., Franklin, MA, USA). NO_3_^−^–N and NH_4_^+^–N were extracted at a soil/water ratio of 1:10 with 2 M KCl solution at 25 °C, and NO_3_^−^ and NH_4_^+^ concentrations were determined using a smart Continuous Flow Analyzer (Smart Chem 140, Shenzhen, China).

### 2.7. Statistical Analysis

Analysis of plant and soil data was carried out using ANOVA. To determine the significance among the treatments, F-test was used, and to find the significance of difference between the means of 2 treatments, the least significant difference (LSD) test was used. LSD, F-test, and ANOVA were analyzed using “Statistix 8.1.1, 2008” and MS Excel, 2019. IBM SPSS Statistics 20 was used for Pearson’s correlation analysis. The heatmaps were created on the R-platform using the “ggplot2” package (version 3.5.1.).

## 3. Results

### 3.1. Plant Growth Parameters

Compared with the control, NPK+RC, NPK+B, NPK, and RC treatments significantly (*p* ≤ 0.05) improved the no. of leaves per plant^−1^ and the plant height (Table 2). Compared with the NPK+B, NPK, and RC treatments, the no. of leaves per plant^−1^ and plant height were also significantly (*p* ≤ 0.05) increased by the NPK+RC treatment. The highest no. of leaves per plant^−1^ and plant height were observed in NPK+RC, followed by the NPK+B and NPK treatments. There was no statistically significant difference between NPK and NPK+B. In addition, the application of chemical fertilizers with rapeseed cake significantly improved the stem diameter of tea plants compared with the NPK+B, NPK, RC, and control treatments. The NPK+RC treatment resulted in the thickest stem diameter, followed by the NPK+B and NPK fertilizers, and the CK treatment resulted in the thinnest stem diameter. No significant difference was recorded between the NPK and RC treatments. The plant height, number of leaves per plant^−1^, and mid stem diameter under the NPK+RC treatment increased by 47.1%, 58.6%, and 28.1%, respectively, compared with the CK (Table 2).

In this study, it was found that the organic and inorganic fertilization treatments significantly improved the leaf fresh weight per plant^−^^1^ (66.3% and 54.3%) and the stem fresh weight per plant^−^^1^ (61.9% and 53.5%), respectively, compared to the control (CK). Compared to the RC treatment, the NPK+RC treatment significantly (*p* ≤ 0.05) improved the leaf and stem fresh weights per plant by 43.8% and 36.6%, respectively (Table 2). The highest leaf and stem fresh weights per plant^−^^1^ were noted in NPK with RC fertilization, followed by the NPK+B treatment, while the control treatment had the lowest leaf and stem fresh weights per plant^−1^.

### 3.2. Catechin Contents in Tea Young Shoots, Stems, and Roots

**Figure 2:**
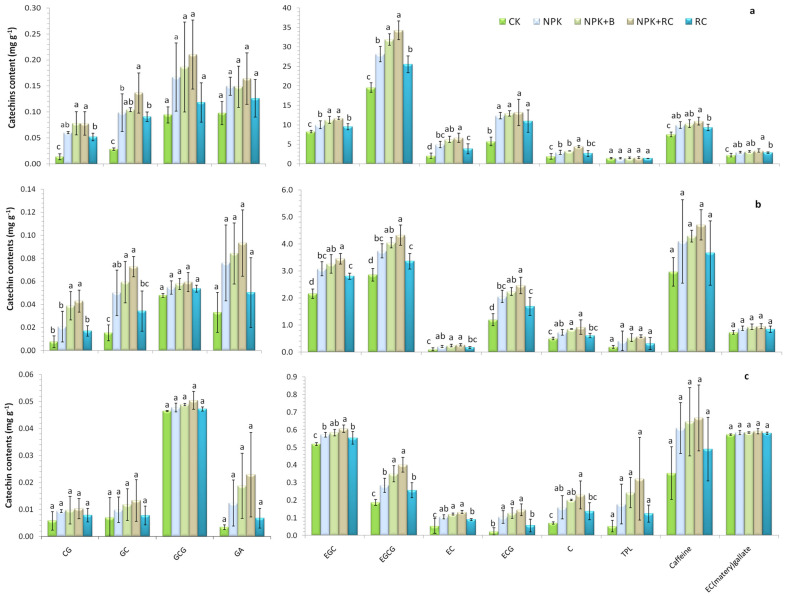
Effect of integrated fertilization on catechin contents (mg g^−1^) in (**a**) young shoots (2 leaves and 1 bud), (**b**) stems, and (**c**) roots of tea (Longjing 43), as determined by HPLC. Gallocatechin (GC), Epicatechin (EC), Gallocatechin Gallate (GCG), Gallic Acid (GA), Catechin Gallate (CG), Epigallocatechin (EGC), Epigallocatechin Gallate (EGCG), Catechins (C), Epicatechin Gallate (ECG). Different lowercase letters indicate significant differences among different treatments at *p* ≤ 0.05, and vertical bars represent the standard deviation of the mean (*n* = 4).

The catechin compositions determined by HPLC in the young shoots (two leaves and one bud), stems, and roots are presented in Figure 2a–c. Various catechin contents were detected in the roots, stems, and young shoots, namely CG, GC, GCG, GA, EGC, EGCG, EC, ECG, C, and EC (matery) gallate; caffeine (CF); and total polyphenol (TPL). It was found that the various catechin contents in different parts of the tea plant were in the order of young shoots> stems >roots. The catechin contents in the roots were significantly lower than those in the other parts of the tea plant. Integrated fertilization had a significant effect on the catechin contents (CG, GC, EGC, EGCG, EC, ECG, EC (matery) gallate, and CF) in the young shoots of tea except GCG, GA, and TPL. The highest catechin contents (CG, GC, EGC, EGCG, EC, ECG, C, EC (matery) gallate, and CF) were recorded in the NPK+RC treatment (0.08, 0.14, 11.7, 34.3, 6.7, 13.2, 4.4, 3.4, and 11.0 mg g^−1^) and the NPK+B treatment (0.08, 0.10, 11.3, 31.9, 6.3, 13.0, 3.3, 3.2, and 10.3 mg g^−1^), while the lowest catechin contents were recorded in the control (0.01, 0.03, 8.3, 19.6, 2.09, 5.7, 1.9, 2.2, and 7.6 mg g^−1^). There was a non-significant difference between the NPK+RC and NPK+B treatments in the majority of catechin contents in young shoots. In the tea stems, the catechin contents (CG, GC, EGC, EGCG, EC, ECG, and C) were significantly affected by organic and inorganic fertilizers, while GCG, GA, TPL, EC (matery) gallate, and CF were not significantly affected by integrated fertilization. The maximum catechin concentrations were established in NPK+RC, while the minimum catechin concentrations were found in the control treatment. The catechin contents between the NPK+RC and NPK+B treatments were not significant (specifically, EGC, EGCG, EC, ECG, and C). The catechin contents of the roots were significantly affected by integrated fertilization, while organic and inorganic fertilization had non-significant effects on the roots’ CG, GC, GCG, GA, EC (matery) gallate, TPL, and CF contents. The concentrations of catechin (EGC, EGCG, EC, ECG, and C) in the NPK+RC treatment were 17%, 115%, 147%, 555%, and 227% higher, and in the NPK+B treatment, they were 13%, 87%, 125%, 473%, and 186% higher than the control treatment, respectively, in the tea roots.

### 3.3. Targeted Metabolites’ Amino Acid Concentrations in Tea Young Shoots, Stems, and Roots

**Figure 3:**
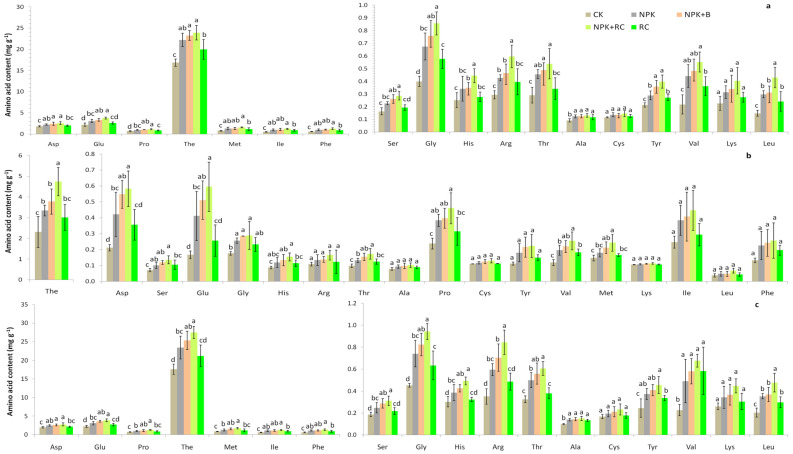
Effect of integrated nutrient management on amino acids (mg g^−1^) in (**a**) young shoots (2 leaves and 1 bud), (**b**) stems, and (**c**) roots of tea (Longjing 43), as assessed by HPLC. Aspartate (Asp), Glutamate (Glu), Proline (Pro), Theanine (The), Isoleucine (Ile), Methionine (Met), Phenylalanine (Phe), Glycine (Gly), Serine (Ser), Histidine (His), Threonine (Thr), Arginine (Arg), Alanine (Ala), Tyrosine (Tyr), Cysteine (Cys), Valine (Val), Leucine (Leu), Lysine (Lys). Different lowercase letters indicate significant differences among different treatments at *p* ≤ 0.05, and vertical bars represent the standard deviation of the mean (*n* = 4).

The concentrations of different free amino acids in the young shoots, stems, and roots of tea (Longjing 43), due to different organic and inorganic fertilizer management methods, are given in Figure 3a–c. It was established that Thea (49–65%), Glu (3–10%), Asp (4–7%), and Pro (2–6%) were the major amino acids in the tea samples. Among the 18 amino acids in the tea young shoots and roots, 16 amino acids (Pro, Ala, Thr, Asp, His, Ser, Gly, Glu, Arg, The, Tyr, Met, Val, Leu, Ile, and Phe) were significantly affected by integrated nutrient management, while 2 amino acids (Cys and Lys) were not significantly affected. The concentrations of amino acids (Asp, Ser, Glu, Gly, His, Arg, Thr, Ala, Pro, The, Tyr, Val, Met, Ile, Leu, and Phe) in the NPK+RC treatment were 41%, 75%, 72%, 114%, 77%, 103%, 85%, 42%, 63%, 42%, 86%, 154%, 109%, 138%, 19%, and 112% higher than the control, and in the NPK+B treatment, they were 33%, 60%, 52%, 90%, 38%, 58%, 68%, 36%, 49%, 38%, 68%, 122%, 72%, 106%, 111%, and 81% higher compared to the control treatment, respectively, in the young shoots. In the roots, the maximum amino acid concentrations were observed in NPK+RC, followed by NPK+B and NPK fertilization, and the minimum amino acid concentrations were observed in the control treatment. In the stems, integrated nutrient management had a significant effect on the amino acid concentrations (Ser, Glu, Thr, Gly, Asp, Pro, His, The, Met, and Val) except for those of the Arg, Ala, Cys, Tyr, Lys, Ile, Leu, and Phe amino acids. The highest amino acid concentrations were observed in the NPK+RC treatment, followed by the NPK+B treatment, while the lowest amino acid concentrations were recorded in the control. There were no significant differences recorded for the amino acid concentrations among the NPK and RC, and among the NPK+B and NPK+RC fertilizer treatments in the roots, stems, and young shoots of green tea. The amino acid concentrations in different parts of the green tea plant were recorded as follows: roots > young shoots > stems.

### 3.4. Chlorophyll Contents of Leaves

**Figure 4:**
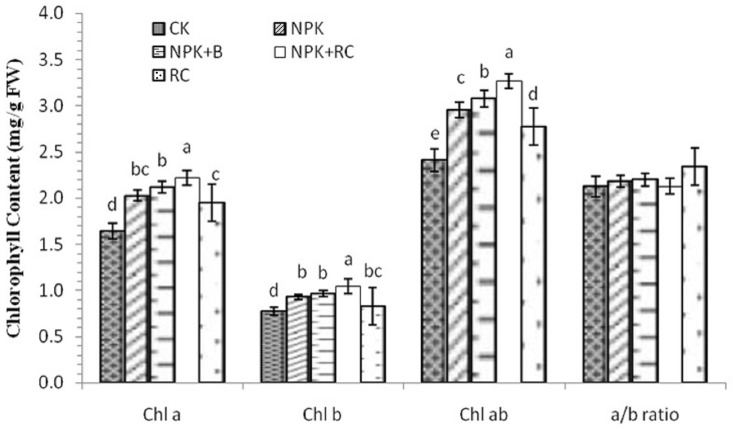
Chlorophyll contents of tea leaves as influenced by integrated nutrient management. Chlorophyll a (Chl a), chlorophyll b (Chl b), chlorophyll ab (Chl ab), chlorophyll a/b ratio (a/b ratio). Different lowercase letters indicate significant differences among different treatments at *p* ≤ 0.05, and vertical bars represent the standard deviation of the mean (*n* = 4).

The effects of integrated fertilization on the chlorophyll contents (Chl) of tea are shown in Figure 4. The research outcome indicated that the concentrations of Chl a, b, and a+b in the tea leaves were significantly affected by organic and inorganic fertilizer management, while for the Chl ratio (Chl a/b), there were no significant differences among all treatments. The chlorophyll a content was higher in the plants treated with NPK+RC, followed by the plants treated with NPK+B integrated fertilization. Furthermore, the lowest chlorophyll a content was found in the control pots and plants treated with RC. The maximum chlorophyll b content was found in the pots treated with integrated fertilization (NPK+RC), followed by those that underwent NPK+B treatment, while the minimum chlorophyll b content was measured in the control treatment pots. There was no significant difference between the NPK and NPK+B fertilizer treatments. The maximum Chl a+b concentration was recorded with the use of mineral fertilizer with RC and mineral fertilizers with biochar. The lowest Chl a+b content was noted in the control treatment. The concentrations of Chl a, b, and a+b in the NPK+RC treatment were 35.5%, 34.1%, and 37.3% higher compared to the control, and 14.3%, 25.8%, and 17.7% higher compared to rapeseed cake, respectively, while NPK+B had 28.9%, 25%, and 27.6% higher Chl a, Chl b, and Chl a+b concentrations compared to the control treatment, respectively.

### 3.5. Soil pH and NH_4_^+^ and NO_3_^−^ Concentrations

The application of integrated nutrient management had a significant effect on the soil pH. Rapeseed cake, alone or in combination, and biochar significantly increased the pH of tea orchard soil (Table 3). The soil pH in the control treatment was 4.41, which is 2.6% higher than that of the NPK treatment. The lowest soil pH (4.30) was recorded in inorganic fertilizers (NPK), while the highest soil pH levels (4.70, 4.64, and 4.55) were recorded in rapeseed cake (RC), NPK+RC, and NPK+B fertilization. Hence, the addition of organic fertilizers, irrespective of their nature, increased the soil pH in acidic soil. In this study, the rapeseed treatments (RC and NPK+RC) significantly changed the pH of the soil from 4.35 to 4.70 and 4.64, respectively.

**Figure 5:**
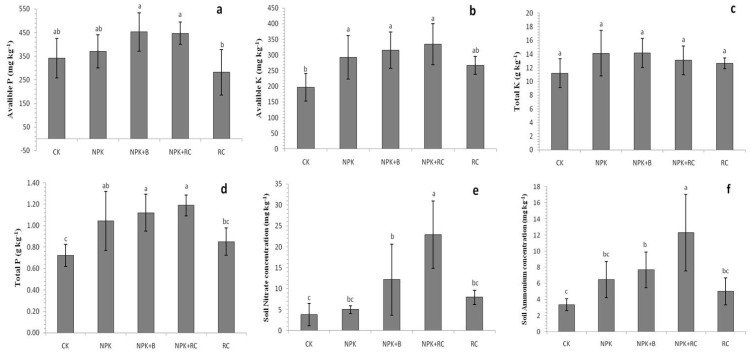
Effect of integrated nutrient management on soil. (**a**) Available P, (**b**) Available K, (**c**) Total P (**d**), Total K (**e**), Nitrate, and (**f**) Ammonium. Different lowercase letters indicate significant differences among different treatments at *p* ≤ 0.05, and vertical bars represent the standard deviation of the mean (*n* = 4).

Significant differences were recorded between the soil’s NH_4_^+^ and NO_3_^−^ concentrations after the application of organic and inorganic fertilizer treatments (Figure 5e,f). The solo application of chemical and organic fertilizers or integrated fertilization application significantly improved the overall soil NH_4_^+^ and NO_3_^−^ concentrations. The maximum concentrations of NH_4_^+^ and NO_3_^−^ were found in NPK+RC, followed by NPK+B and NPK, and the minimum contents of NH_4_^+^ and NO_3_^−^ were found in the control and RC treatments. NPK+RC increased the NO_3_^−^ and NH_4_^+^ concentrations by 504% and 267% compared to the control treatment, and by 188% and 146% compared to the RC treatment, respectively. Similarly, the NPK+B and NPK treatments also improved the NO_3_^−^-N concentration by 221% and 33% compared to the control treatment, while the NH_4_^+^-N concentration showed increases of 129% and 93% in the NPK+B and NPK fertilizer treatments relative to the control.

### 3.6. Soil Micro and Macronutrients

Organic and inorganic fertilizers significantly affected the soil micro- and macronutrients. The application of mineral fertilizers (NPK), along with RC and B amendments, effectively improved the total N, P, and C concentrations of the soil. The potassium content of the soil was not significantly improved by integrated fertilization (Table 3; Figure 5c,d). The highest total N, P, and C contents of the soil were observed in the NPK+RC treatment, while the lowest were recorded in the CK and RC treatments. The NPK+B treatment also improved the total N, P, and C concentrations of the soil. The NPK+RC treatment increased the macronutrient (N, P, and C) concentrations by 43%, 65%, and 31% compared to the control treatment, respectively, followed by the NPK+B treatment. The carbon content in the rapeseed cake was significantly higher than that of the mineral fertilizer (NPK) treatment. The N content was significantly higher in the chemical fertilizers (NPK) compared to the solo application of rapeseed cake fertilizer. Integrated fertilization significantly (*p* ≤ 0.05) affected the C:N ratio of the soil (Table 3). Compared with organic fertilizer alone, integrated fertilization showed a lower C:N ratio. Among the treatments, the C:N ratio trend was as follows: CK > RC > NPK > NPK+B > NPK+RC. The C/N ratios of the NPK+RC and NPK+B treatments were low, indicating that the N content was higher than that of NPK and RC. The rapeseed cake and NPK+RC treatments had higher Mg and Ca contents, followed by the NPK+B treatment. RC improved the Mg and Ca contents by 72% and 100% compared to CK, and by 25% and 60% compared to chemical fertilizer. NPK+RC also improved the Mg and Ca contents by 48% and 88% compared to the control, respectively (Table 3).

An analysis of the soil test data revealed that the available P and K concentrations of the soil responded significantly (*p* ≤ 0.05) to different organic and inorganic fertilizer applications (Figure 5a,b). After the application of treatments, the available P and K concentrations of the soil ranged from 281.9 to 452.8 mg kg^−1^ and from 196.6 to 334.5 mg kg^−1^. The soil’s available P and K concentrations of rapeseed cake with chemical fertilizers (NPK+RC) were significantly higher than those of the soil with the solo application of RC and inorganic fertilizer (NPK). NPK+B and NPK+RC fertilization improved the available P concentrations of the soil by 32% and 31%, while the available K concentrations of the soil improved by 70% and 60% compared to the control treatment, respectively.

This study also demonstrated that organic fertilizers alone or in combination with mineral fertilizer treatments significantly (*p* ≤ 0.05) improved the contents of the soil micronutrients (Mn, Fe, Zn, and Cu) (Table 3). The organic fertilizers had significant high amounts of micronutrients compared to the inorganic fertilizers. Therefore, the highest soil micronutrients (Mn, Fe, Zn, and Cu) were observed in rapeseed cake, followed by the NPK+RC and NPK+B fertilizer treatments, while the lowest soil micronutrients were recorded in the control and chemical fertilizer (NPK) treatments. Rapeseed cake (RC) significantly increased the micronutrients (Fe, Mn, Cu, and Zn) of the soil by 49%, 161%, 112%, and 40% compared to the control, and by 12%, 66%, 82%, and 45% compared to chemical fertilizer treatment, respectively. The soil micronutrients (Fe, Mn, Cu, and Zn) were also improved by NPK+RC (47%, 75%, 45%, and 14%) and NPK+B (26%, 72%, 25%, and 2%) compared to the CK treatment, respectively. There was no significant difference in the soil micronutrients between the NPK+RC and RC treatments. So, the organic fertilization or the incorporation of integration fertilization improved the soil micronutrient statutes in the tea plantations, which promoted tea growth and quality.

### 3.7. Pearson’s Correlation Analysis of Soil Chemical Properties and Plant Growth

**Figure 6:**
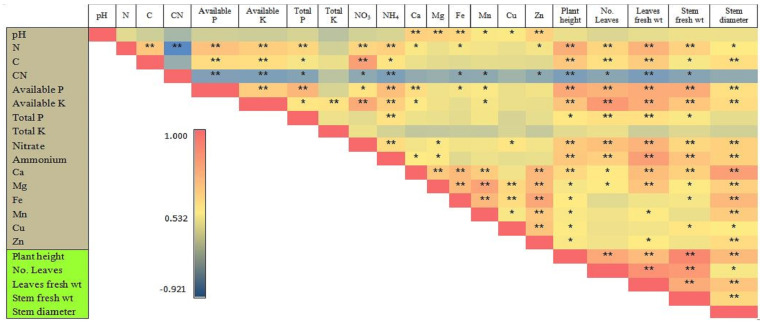
The Pearson’s correlation analysis of soil chemical properties and plant growth. An asterisk indicates the significance of the correlations at *p* levels of ≤0.01 (**) and ≤0.05 (*).

A Pearson’s correlation matrix for the relation of the soil chemical characteristics (pH and soil macro- and micronutrients) and plant growth factors (plant height, no. of leaves per plant, avg. stem diameter, and leaf and stem fresh weights per plant) of tea are presented in Figure 6. Most of the correlations between soil macro- and micronutrients were significantly positively correlated with each other, except for the soil’s C:N ratio, which had significant negative correlations with the plant growth parameters and soil macro- and micronutrients. The soil macro- and micronutrients also had significant positive correlations with the plant growth parameters, except the total K did not have a significant positive correlation with the plant growth parameters; Fe and Cu did not have significant correlations with the no. of leaves per plant and the leaf fresh weight per plant; and Mn and Zn did not have significant correlations with the no. of leaves per plant and the stem fresh weight per plant. The soil pH was significantly positively correlated with the soil macro- and micronutrients (Mg, Ca, Mn, Fe, Zn, and Cu), and among the soil macro- and micronutrients, Ca, Mg, Fe, and Zn had very strong and significant positive correlations with the soil pH. The soil’s total K, P, N, and C did not have a significant correlation with the soil micronutrients, except N, which had a significant positive correlation with Ca, Fe, and Zn. The available P and K had significant correlations with the plant growth parameters; the soil’s total N, C, and P; the soil’s NO_3_^−^ and NH_4_^+^; and the soil’s Ca, Fe, and Mn concentrations. Nitrate was significantly positively correlated with NH_4_^+^, Mg, Cu, and the plant growth parameters. Ammonium was significantly positively correlated with only Ca and Mg and the plant growth parameters. All of the soil micronutrients were strongly and significantly positively correlated with each other’s, except Ca, which was not significantly correlated with the soil Cu. The tea growth variables (plant height, no. of leaves per plant, avg. stem diameter, and leaf and stem fresh weights per plant) were also strongly and significantly positively correlated with each other.

### 3.8. Pearson’s Correlation Analysis of Chlorophyll Content and Soil Chemical Properties with Different Catechin Contents in Tea Young Shoots

**Figure 7:**
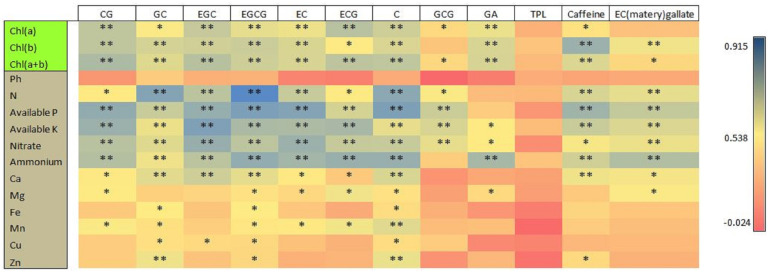
The Pearson’s correlation analysis of chlorophyll content and soil chemical properties with young shoot catechin contents. An asterisk indicates the significance of the correlations at *p* levels of ≤0.01 (**) and ≤0.05 (*).

The leaves’ Chl contents (Chl a, Chl b, and Chl a+b) and the soil chemical properties (total N; available P and K; and NO_3_^−^, NH_4_^+^, Ca, Fe, Mg, Cu, Mn, and Zn) were significantly positively correlated with different catechin contents of young shoots (Figure 7). The Chl contents (Chl a, Chl b, and Chl (a+b)) were significantly positively correlated with all catechin types of young shoots, except GCG and EC (matery) gallate, which were not significantly correlated with Chl b and a, respectively. The TPL is also not significantly correlated with the chlorophyll contents and soil chemical properties. The pH of the soil did not have significant correlations with the different catechin types. The soil macronutrients, including the total N, nitrate, ammonium, and available P and K, were significantly positively correlated with different catechin types, except GCG and GA. The soil macro- and micronutrients (Mg, Ca, Mn, Fe, Zn, and Cu) also had significant positive correlations with some catechin types, and some catechin types had non-significant positive correlations, for example, EGC, GCG, GA, TPL, EC (matery) gallate, and caffeine. Generally, significant positive correlations exist between soil macro- and micronutrients and different catechin types of tea young shoots.

### 3.9. Pearson’s Correlation Analysis of Chlorophyll Contents and Soil Chemical Properties with Different Targeted Amino Acids of Tea Young Shoots

**Figure 8:**
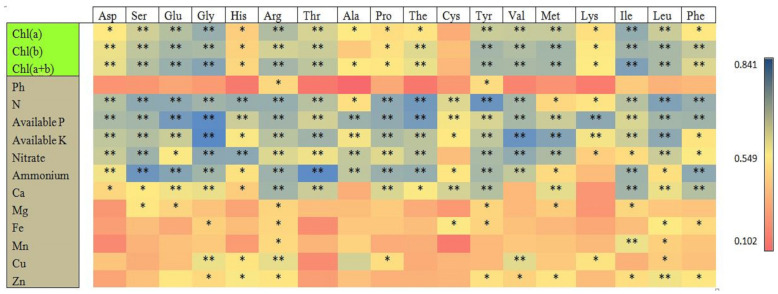
The Pearson’s correlation analysis of chlorophyll content and soil chemical properties with young shoot’s targeted amino acid metabolites. An asterisk indicates the significance of the correlations at *p* levels of ≤0.01 (**) and ≤0.05 (*).

The correlation matrix of the leaf chlorophyll contents and soil macro- and micronutrients with different targeted amino acid metabolites of tea young shoots is shown in Figure 8. The chlorophyll contents and soil macro- and micronutrients were significantly positively correlated with the different amino acids of the tea young shoots. Ala was correlated with Chl a, Cys was correlated with Chl a, and Chl b and Chl a+b were not significantly correlated, respectively. The soil macronutrients were significantly positively correlated with most of the targeted amino acids of the young shoots compared to the soil micronutrients. The soil pH only had a significant positive correlation with Arg and Tyr. The nitrate and ammonium of the soil did not have a significant correlation with the Cys and Lys amino acids of the tea young shoots, respectively. In the case of micronutrients, a significant positive correlation existed between the soil micronutrients and 17–39% of different targeted amino acids, except Ca and Zn, which had significant positive correlations with 83% and 50% of different amino acids in the tea young shoots, respectively.

## 4. Discussion

### 4.1. Effects of Different Fertilization Amendments on Plant Growth

Compared with the CK, the NPK+RC and NPK+B fertilizations significantly improved the plant height, stem circumference, leaf number, and fresh biomasses of the leaves and stems. The application of integrated fertilization has been reported to promote plant growth. For example, Rafael et al., 2019 [35] reported that the use of NPK+B resulted in a 4–5-fold increase in the cowpea biomass yield compared to the control, and it resulted in a 70% improvement compared to chemical fertilizer (NPK) treatments. Saha et al., 2019 [37] exemplified the synergetic effects of inorganic fertilizers and biochar on pasture yield. Soil characteristics, such as the microbial activity, pH, and CEC, were significantly improved by biochar [33,35]. After the application of biochar and RC to inorganic fertilizers, the enhancement of tea growth factors may be connected to the enhancement of the soil chemistry (pH and nutrient concentration) and microbial properties (soil fungal and bacterial diversity index). This finding is in accordance with the study by Bass et al., 2016 [45], which may indicate that organic fertilizers do not meet the nutritional needs of tea plants during growth. Integrated fertilization utilizes chemical fertilizers as a nutrient source and biochar as a soil amendment [33]. In addition, many studies have reported that B improves the nutrient use efficiency of mineral fertilizers [37,46]. We think this may be related to the adsorption of macronutrients (P, K, and N) by RC and B, which leads to a sustained released of nutrients and provides more macro- and micronutrients for tea plant growth. Particularly in sub-tropical tea-growing regions with higher rainfall and lower pH levels, organic fertilizers play a vital role in sustaining soil macro- and micronutrients and decreasing nutrient loss and the leaching of chemical fertilizers [47]. The increase in Casuarina height after biochar application can be attributed to augmented plant nutrient availability, such as potassium and phosphorus, and improved soil physical properties, such as reduced BD and improved soil water storage [48,49]. Nevertheless, research on the effects of biochar on tree growth remains prominent [50]. Research has further shown that soil improved with 3% biochar resulted in the highest increases in the leaf count and plant height.

The favorable effect of optimal nutrient levels obtained through inorganic and organic nutrient sources on the increasing leaf number per plant^−1^ could be one of the reasons for the higher leaf fresh weight per plant^−1^. Under chemical fertilization, an insufficient nutrient supply throughout the growing phase may limit the growth rates of the leaves, thereby limiting the number of leaves per plant^−1^ due to low inadequate cell expansion or photosynthesis rates or both [51]. Ramana et al., 2002 [52] also reported that inadequate and unbalanced nutrient supply are often the main reasons why plants are unable to achieve their potential growth. The use of integrated fertilization increases the intrinsic nutrient supply capacity of the soil to macro- and micronutrients [53] and improves the soil physical properties, thereby promoting better rooting, improving crop nutrient uptake, and promoting plant growth.

### 4.2. Effect of Integrated Fertilization on Catechin Contents in Roots, Stems, and Young Shoots of Tea

Catechin is the main secondary metabolite in Chinese tea, which has a significant impact on the tea taste and quality. In this research, the concentrations of various catechin components, namely CG, GC, GCG, GA, EGC, EGCG, EC, ECG, C, and EC (matery) gallate; caffeine (CF); and total polyphenol (TPL) were determined in the young shoots, stems, and roots of tea plants under different integrated nutrient management strategies. The tea young shoots (two leaves and one bud), followed by the stems, had the highest catechin contents, while the tea roots had the lowest catechin contents, which is in accordance with prior findings [54]. Due to the higher availability of micro- and macronutrients, the application of mineral fertilizers (NPK) and organic fertilizers (RC and B) increases the content of catechins in tea. Organic fertilizer improved the biological, chemical, and physical soil properties, thereby augmenting the yield and quality of tea [4]. Bagchi et al., 2022 [55] also found maximum contents of catechins in organic-based fertilizers compared to inorganic fertilizers and the control. Both the EC and ECG catechins belong to the class of dehydroxylated catechins, signifying that dehydroxylated catechin biosynthesis plays a key role in the root system, whereas EGC and EGCG were the major catechin constituents in the leaves (63%) and stems (64% in total).

Catechins are synthesized via the flavonoid and phenylpropanoid biosynthetic pathways. However, the individual catechins’ exact biosynthetic pathways remain unknown. Stafford, 1990 [56] stated that C is an isomeric transition for the conversion of (+)-dihydroquercetin to EC. However, recent studies have found that the synthesis of EC and C is catalyzed by anthocyanin synthase (ANS) and leucocyanin reductase (LAR) from leucocyanin, respectively [54]. In addition, Wellmann et al., 2006 [57] reported that ANS can convert C into two major products and one minor product, suggesting that C may be involved in multiple biosynthetic pathways as an intermediate. The properties of catechins vary. For example, compared to other catechins, the (+)-catechin (C) significantly reduces the bitterness and astringency of tea.

### 4.3. Effect of Integrated Fertilization on Amino Acid Concentration in Tea Young Shoots, Stems, and Roots

In this research, compound fertilization (NPK+RC and NPK+B) significantly improved the amino acid contents of the young shoots (two leaves and one bud), roots, and stems. The amino acid content was higher in the roots compared to the stems. Former studies have reported that many amino acids were synthesized primarily in tea roots and then transported from the roots to the stems [58,59]. In this research, the application of organic-based fertilization enhanced nutrient uptake in tea a process that may affect the production of amino acid contents [60]. Consequently, integrated fertilization may improve amino acid synthesis in tea. In addition, rapeseed cake and biochar can increase the soil water holding capacity, thereby improving the leaf water fluidity and increasing the production of amino acids [37]. More importantly, B with NPK fertilizers can interact with the roots and influence the production of root secondary metabolites, finally influencing the quality of tea [22,36]. Organic fertilizers provide micronutrients that help in the improvement of tea quality and different amino acid concentrations with inorganic fertilizer application. In addition, integrated fertilization affected the soil microbiota, thereby increasing the amino acid contents of the tea. Studies have shown that microorganisms have positive correlations with tea yield and quality. Several genera, such as Rhodosporidium, Sphingomonas, Rhodanobacter, Chloridiums, Devosa, and Inocybes, have antecedently been reported to be participating in plant growth promotion and nutrient cycling [61,62]. Among the various metabolites, amino acids contribute a lot to the quality of tea. Studies have shown that nitrogen form and nitrogen levels significantly affect amino acid metabolism, thereby regulating the amino acid contents in the tea roots and aerial parts. There is growing evidence that nitrogen forms alter the amino acid content in tea tree leaves and roots [21,63,64]. Huang et al., 2018 [65] showed that providing different forms of nitrogen to tea plants significantly increased the Glu, Thea, and Pro contents in tea shoots compared to the control group. Through theanine synthetase, theanine is synthesized from glutamic and ethylamine acids. The decarboxylase of alanine in tea produced ethylamine. The N in these organic compounds comes from NO_3_^−^ or NH_4_^+^. Tea plants prefer ammonium ions as inorganic N sources for growth [21].

Present studies have shown that amino acids provide different characteristics for the taste quality of tea infusion [66,67]. Thea, the most abundant amino acid, imparts complex flavors, including umami, bitterness, and sweetness, and the intensity level of the taste increase with an increasing Thea concentration [6,66]. Glu and Asp also contribute to the umami flavor. Gly, Ala, Thr, Pro, and Ser produce a sweet taste, while His, Ile, Arg, Met, Phe, Leu, Val, and Trp produce a bitter taste. The umami taste of tea infusion is primarily attributed to amino acid, especially Thea [68].

### 4.4. Chlorophyll Content of Leaves as Affected by Organic and Inorganic Fertilizer Management

The maximum concentrations of Chl a, b, and Chl a+b were found in the NPK+B and NPK+RC treatments, while the control and RC treatments had a minimum chlorophyll concentration. Similar studies were conducted at the seedling stage of peanuts, where the chlorophyll content increased with the application of biochar-based fertilizers [69]. Another similar result was found in soybean [70], sorghum, and maize plants [71], where inorganic fertilizers added to organic fertilizers increased the total chlorophyll content. The application of NPK-free organic fertilizer does not adequately provide the nutrients required for Chl biosynthesis. However, the addition of biochar and rapeseed cake in NPK applications significantly improved nutrient uptake, thereby optimizing chlorophyll biosynthesis. Large amounts of Chl are needed to maintain photosynthetic pigments and synthesized enzymes involved in tea growth, biomass, and quality. Higher photosynthetic rate mechanisms increase the chlorophyll concentration of tea, which induces higher plant growth.

The chemical structures of chlorophyll a (C_55_H_72_O_5_N_4_Mg) and chlorophyll b (C_55_H_70_O_6_N_4_Mg) have different functional groups. In addition, the color of Chl b is light green, and Chl a has a dark green color. The light energy is converted into chemical energy by the principal pigment, Chl a. Chl b acts as a co-pigment and plays an indirect role in photosynthesis by transporting the light it absorbs to Chl a [72]. Green tea products and the green color of fresh tea leaves are created by the Chl compound. Water-soluble green pigments, chlorophyllides, and chlorophyll derivatives are derived from chlorophyll through the catalysis of chlorophyllase, which is dissolved during the tea brewing process, resulting in green tea-colored infusions [73]. The chlorophyll content gives green tea products a grassy flavor [74].

### 4.5. Effect of Integrated Fertilization on Soil pH and NH_4_^+^ and NO_3_^−^ Concentrations

In this research, the soil pH was significantly influenced by combined fertilization, where the soil pH of inorganic fertilizer (NPK) decreased; rapeseed cake alone or in combination with inorganic fertilizer (NPK+RC) increased the soil pH; and the incorporation of biochar and chemical fertilizer (NPK+B) also improved the soil pH due to its liming properties. These results show that organic fertilizers have greater potential to increase the soil pH than nitrogen, phosphorus, and potassium fertilizers. This means that organic fertilizers can be used as good materials for improving acidic soils. The results are consistent with those of many similar studies, such as Yang et al., 2021 and Xie et al., 2021 [75,76], who noted that the pH of acidic soils increases with the incorporation of organic fertilizers.

The nationwide average pH of tea garden soils is 4.68, and the soil pH in different provinces in China varies from 3.96 to 5.48 [22]. Tea garden soils are acidic, with a pH of about 4.0 to 5.5, unlike other crops [77,78]. When the pH of the soil is less than 4, it is not conducive to the growth and quality of tea plants [79]. Therefore, increasing the soil pH is essential to improving tea soil fertility. In general, the addition of biochar reduces stress on plants due to its lime properties [80]. Similarly, Zhang et al., 2019 and Saha et al., 2019 [37,81] also indicated that the use of biochar (B) or biochar-based fertilizers increases the soil pH. Therefore, applying RC and biochar plus mineral fertilizer is a suitable way to alleviate acidification in tea orchard soil.

Combined fertilization increased the soil NO_3_^−^ and NH_4_^+^ concentrations. The application of RC and B with NPK significantly increased the soil NO_3_^−^ and NH_4_^+^ contents, while the NO_3_^−^ and NH_4_^+^ contents of the RC and control treatments were lower. The content of ammonium in soil was significantly higher than that of nitrate, and the content of ammonium in RC and biochar with chemical fertilizer treatments was significantly higher than that in the mineral fertilizer treatments (Figure 5e,f). Therefore, RC is more beneficial to the growth of tea plants than mineral fertilizers that prefer NH_4_^+^. At the same time, the nitrate content in the overflow water was higher than the ammonium content. Ammonium in soil is generally adsorbed by the soil organic matter and soil particles, and ammonium is rapidly converted to nitrate under aerobic soil conditions [76]. The utilization of cow manure biochar (CMB) and straw biochar+ fertilizer (RSBF) increased the NH_4_^+^ content by 45.1% and 36.5%, respectively, compared to chemical fertilizers [82].

### 4.6. Effect of Integrated Fertilization on Soil Macro and Micronutrients

The application of B and RC with chemical fertilizers (NPK+B and NPK+RC) significantly improved soil macro- and micronutrient statuses. Regarding the soil nutrient status, chemical fertilizer and organic fertilizer jointly improved plant growth and significantly increased the N, P, and K contents in the soil. Organic amendments using chemical fertilizers may result in higher microbial activity and nutrient availability compared to no fertilization (controls) and mineral fertilization [61,62,83]. When organic fertilizer is applied together with chemical fertilizers, the application of organic amendments increases the soil’s N, P, and K concentrations [83]. Organic fertilizers have a greater impact on the soil quality than chemical fertilizers, thereby improving nutrient release and their availability to tea plants [84]. Rapeseed cake significantly improved the exogenous input of the soil OM, which is more likely to retain nutrients in a form that is more easily absorbed by plants for a longer period of time. The nutrients in chemical fertilizers are already mineralized, which provides a ready source of nutrients for the soil. It is implied that the nutrient release time of NPK fertilizers was short because the leaching of nutrients from soils treated with NPK fertilizers may be higher than that of soils treated with organic fertilizers. Several studies have reported a longer residual effect of organic fertilization on soils [85].

Our results also indicated that the soil’s available contents of K, P, N, Ca, etc., were significantly improved by the NPK+B treatment, which is also consistent with previous studies [29,33,75]. The available soil C content has been shown to play a critical role in conserving the fertility of soil. Biochar adsorbs OM and promotes the establishment of humus soil, which increases the amount of available soil carbon and nitrogen [86]. As for other nutrients, biochar itself carries certain nutrients, such as phosphorus, potassium, and calcium, which can directly provide nutrients. In addition, biochar is able to interact with soil and indirectly affect the availability of nutrients in soil through adsorption, precipitation, and dissolution [87]. To store nutrients in soil, biochar plays an important role in reducing nutrient leaching and nutrient loss from inorganic fertilizers [47,75]. These outputs may be due to the higher macronutrient content in the NPK+B treatment. Similar results have previously been reported; biochar-based fertilizers produce a higher soil nutrient value than biochar alone [29,81].

Synthetic chemical fertilizers are primarily used as nitrogen sources for plant growth, while RC fertilizer not only supplies nitrogen but also helps to maintain the carbon content in the soils. Therefore, chemical fertilizer and RC organic fertilizer have different effects on the soil’s carbon and nitrogen cycles in tea gardens. Compared with rapeseed cake and biochar alone, the combined application of chemical and organic fertilization has a lower C:N ratio. However, the CK and NPK treatments had higher C:N ratios. The change in the proportion of carbon and nitrogen sources in the soil after fertilization may be due to the fact that the chemical fertilizer is a synthetic fast-acting N fertilizer, which is primarily used to supply the nitrogen needed by the plant, so little carbon is input. Rapeseed is a kind of biomass fertilizer that also contains a large quantity of OM, which provides a huge quantity of carbon to the soil. As a slow-release fertilizer, RC has a relatively small and slow nitrogen input. Similar research has also shown that organic N in organic fertilizer is more effective than chemical nitrogen in synthetic fertilizer, particularly in low-yield agricultural production systems [76,88]. The lower C:N ratio of the soil treated with integrated fertilization may be attributed to higher nitrogen availability due to the accumulation of chemical and organic fertilizers and their retention in the soil, which is associated with lower nitrogen loss rates than carbon loss rates during organic matter degradation [89]. Soil organic carbon was significantly improved by organic amendments, which has a significant influence on soil micro- and macronutrient availability, microbial activity, and nutrient uptake, potentially altering the C:N ratio. However, the incorporation of external organic matter with higher C:N ratios may also speed up the mineralization of existing OM, releasing the N trapped in existing OM—a process known as the priming effect [90]. These outcomes are confirmed by former studies [83].

The application of integrated fertilization improved NUE and the recovery of micro- and macronutrients. The improvement of OM may be due to the application of organic fertilizers, which improve soil properties and plant growth. Adediran et al., 2005 [91] reported that plots treated with organic fertilizers increased organic carbon by 11–20%, calcium by 26–96%, magnesium by 6–19%, and zinc by 4–8% compared with chemical fertilizers. The application of integrated nutrient management increases the content of soil micronutrients compared with the solo application of chemical fertilizers. Similarly, organic fertilizer sources also contributed to the supply of soil macro- and micronutrients through mineralization processes and improved the soil physicochemical and microbial properties. Furthermore, these fertilizers are able to gradually release nutrients (macro and micronutrients) and make them available to plants throughout the growing period [91]. Thus, the application of integrated fertilization was considered to be a good choice for promoting nutrient recovery and plant growth [83].

## 5. Conclusions

This study investigated the effects of integrated fertilization on soil chemistry, tea growth and quality, and the relationship between soil chemistry and tea growth and quality. Studies have shown that NPK+RC and NPK+B amendments increase the soil pH, macronutrients, and micronutrients, especially soil-available nutrients. Additionally, changes in the soil chemistry caused by integrated fertilization were key factors in improving the growth parameters of the tea plant. Furthermore, integrated fertilization significantly improves the quality of tea compared to the solo application of mineral fertilizers or organic fertilizers. Combined fertilization also significantly improved the Chl concentrations (Chl a, Chl b, and Chl a+b). The growth parameters and quality of tea were significantly positively correlated with soil macronutrients (N, P, K, Ca, and Mg), nitrate, ammonium, and soil micronutrients (Cu, Mn, Fe, and Zn). The chlorophyll content was also significantly positively correlated with tea quality and growth. The present study suggested that, compared with organic or inorganic fertilizer alone, integrated fertilization (NPK+RC and NPK+B) shows better performance in promoting soil properties and tea growth and quality, and it has important potential to improve the chemical properties of degraded tea garden soil and further improve the growth and quality of green tea.

## Figures and Tables

**Table 1:** Basic characteristics of tea orchard top rhizosphere soil (0–20 cm soil depth).

Soil Properties	Means
Soil texture	Clay
Clay (%)	63.0 ± 2.2
Sand (%)	9.0 ± 0.2
Silt (%)	28.0 ± 1.0
EC (μS cm^−1^)	108.0 ± 4.6
pH	4.4 ± 0.01
Available K (mg kg^−1^)	114.0 ± 2.7
Available P (mg kg^−1^)	30.0 ± 0.06
NO_3_^−^ (mg kg^−1^)	16.0 ± 0.4
NH_4_^+^(mg kg^−1^)	33.0 ± 2.2
P (g kg^−1^)	0.8 ± 0.01
N (g kg^−1^)	2.7 ± 0.01
K (g kg^−1^)	12.0 ± 0.1
C (g kg^−1^)	18.0 ± 0.02

Mean ± standard deviation.

**Table 2:** Effect of integrated fertilization on plant height, no. of leaves per plant^−1^, leaf and stem fresh weights, and stem diameter.

Treatments	Plant Height (cm)	No. of Leaves per Plant^−1^	Leaf Fresh Weight (g Plant^−1^)	Stem Fresh Weight (g Plant^−1^)	Stem Mid Girth (mm)
CK	22.63 ± 1.80 c	46.0 ± 8.2 c	6.22 ± 1.23 d	2.73 ± 0.95 c	2.18 ± 0.09 c
NPK	32.75 ± 3.30 b	71.0 ± 22.8 b	12.04 ± 0.80 bc	4.65 ± 1.50 bc	2.76 ± 0.17 b
NPK+B	34.00 ± 4.08 b	80.0 ± 19.9 b	13.62 ± 1.52 b	5.87 ± 1.48 ab	2.83 ± 0.19 ab
NPK+RC	42.75 ± 4.57 a	111.0 ± 10.5 a	18.47 ± 3.98 a	7.16 ± 1.32 a	3.03 ± 0.10 a
RC	30.50 ± 6.66 b	58.0 ± 9.1 bc	10.38 ± 1.28 c	4.54 ± 1.23 bc	2.58 ± 0.14 b

Control (CK); Chemical Fertilizers (NPK); Chemical Fertilizer + Biochar (NPK+B); Chemical Fertilizer + Rapeseed Cake (NPK+RC); Rapeseed Cake (RC). Data are shown as means ± standard deviation, and different lowercase letters indicate significant differences among different treatments at *p* ≤ 0.05 levels in the same line (LSD test).

**Table 3:** Effect of organic and inorganic fertilization on soil pH and macro and micronutrients.

Treatments	pH	N (%)	C (%)	CN Ratio	Ca (mg kg^−1^)	Mg (mg kg^−1^)	Fe (mg kg^−1^)	Mn (mg kg^−1^)	Cu (mg kg^−1^)	Zn (mg kg^−1^)
CK	4.41 ± 0.09 bc	0.19 ± 0.005 c	1.56 ± 0.03 c	9.57 ± 0.33 a	154.66 ± 54 c	24.73 ± 3.4 b	267.63 ± 31 b	10.48 ± 2.5 c	1.01 ± 0.06 b	1.74 ± 0.13 b
NPK	4.35 ± 0.03 c	0.21 ± 0.03 bc	1.61 ± 0.06 c	8.78 ± 0.93 ab	202.57 ± 41 bc	34.00 ± 5.8 ab	358.39 ± 32 a	16.48 ± 2.3 bc	1.18 ± 0.11 b	1.68 ± 0.22 b
NPK+B	4.55 ± 0.17 ab	0.23 ± 0.02 ab	2.01 ± 0.07 a	7.61 ± 0.96 b	275.61 ± 34 ab	30.70 ± 3.4 ab	336.12 ± 24 ab	17.99 ± 8.6 b	1.27 ± 0.21 b	1.70 ± 0.08 b
NPK+RC	4.64 ± 0.13 a	0.26 ± 0.03 a	2.05 ± 0.04 a	7.74 ± 0.19 b	290.44 ± 43 ab	36.68 ± 11.5 a	393.99 ± 40 a	18.39 ± 4.3 b	1.46 ± 0.43 b	1.98 ± 0.24 b
RC	4.70 ± 0.09 a	0.20 ± 0.01 c	1.77 ± 0.06 b	7.69 ± 1.08 b	324.30 ± 116 a	42.45 ± 10.9 a	399.85 ± 100 a	27.39 ± 3.8 a	2.14 ± 0.59 a	2.43 ± 0.34 a

Control (CK); Chemical Fertilizers (NPK); Chemical Fertilizer + Biochar (NPK+B); Chemical Fertilizer + Rapeseed Cake (NPK+RC); Rapeseed Cake (RC). N: Nitrogen; C: Carbone; Mg: Magnesium; Ca: Calcium; Fe: Iron; Cu: Copper; Mn: Manganese; and Zn: Zinc. Data are shown as means ± standard deviation, and different lowercase letters indicate significant differences among different treatments at the *p* ≤ 0.05 level in the same line (LSD test).

## Data Availability

Data will be available upon request.
